# Heat shock protein 27 deficiency promotes ferrous ion absorption and enhances acyl-Coenzyme A synthetase long-chain family member 4 stability to promote glioblastoma cell ferroptosis

**DOI:** 10.1186/s12935-023-02848-3

**Published:** 2023-01-13

**Authors:** Kai Zhang, Yue Wu, Guangliang Chen, Hao Wang, Yongsheng Liu, Youxin Zhou

**Affiliations:** Department of Neurosurgery, Laboratory of Brain and Nerve Research, The First Affiliated Hospital of Soochow University, Suzhou, 215006 Jiangsu China

**Keywords:** HSP27, ACSL4, GBM, Fe^2+^, SUMO, ROS, Ferroptosis

## Abstract

**Background:**

Glioblastoma is one of the malignant tumors of the central nervous system with high lethality, high disability and low survival rate. Effective induction of its death is one of the existing challenges. In recent studies, heat shock protein 27 (HSP27) has been shown to be associated with ferroptosis; therefore, targeting HSP27 may be a potential therapeutic approach for GBM.

**Methods:**

Immunohistochemistry and western blot analysis were used to detect the expression of HSP27 in GBM tissues. CCK8, plate clone formation assay, EdU proliferation assay for cell proliferation ability, PI, LDH release assay for cell viability. Reactive oxygen, iron levels, and mitochondrial potential for HSP27 silencing were assayed for ferrotosis in vitro. Western blotting and IP were used to verify the relationship between HSP27 and ACSL4. The effect of knockdown of HSP27 on tumor growth capacity was assessed in an intracranial xenograft model.

**Results:**

HSP27 was significantly highly expressed in GBM. In vitro experiments, knockdown of HSP27 significantly induced ferroptosis in GBM cells. IP and western blot demonstrated a sumo-ization link between HSP27 and ACSL4. In vivo experiments, HSP27 deficiency retarded tumor growth rate by promoting ferroptosis.

**Conclusions:**

HSP27 deficiency promotes GBM ferroptosis. Targeting HSP27 may serve as a new direction for GBM treatment.

## Introduction

Glioblastoma (GBM) is a World Health Organization grade IV central nervous system tumor [1] characterized by a high degree of malignancy, high mortality, high recurrence rate, and short survival. Despite early surgical intervention and postoperative chemoradiation therapy, the quality of life of patients with GBM remains poor and the median survival is generally not more than 15 months [2]. GBM is associated with chemotherapy resistance, pro-angiogenesis properties, and is extremely epigenetically unstable [3] [4]. Compared to normal cells, the iron content and iron-related protein expression of GBM cells vary greatly [5]. Targeting iron-associated protein expression, or altering intracellular iron levels, is considered a viable strategy for treating tumors [6, 7]. Previous studies have found that inducing ferroptosis in GBM cells can exert better therapeutic effects [8]. Therefore, understanding the molecular mechanisms involved in ferroptosis involved in GBM could be beneficial for the treatment of the disease.

Ferroptosis is a newly discovered type of programmed cell death, which is different from traditional apoptosis, necrosis, and autophagy [9]. It is characterized by cell death due to increased intracellular free ferrous ions, peroxidation of membrane lipid polyunsaturated fatty acids (PUFA), and decreased synthesis of glutathione peroxidase 4 (GPX4), leading to cell death [10]. There are almost no organisms that do not contain iron, and compared with normal cells, tumor cells require a lot of iron to maintain growth, reproduction, migration, and invasion [11]. Iron deficiency can lead to a decrease in cell viability, and too much iron can produce more toxic hydroxyl radicals with hydrogen peroxide through the Fenton reaction, triggering cellular lipid peroxidation [12]. This suggests that tumor cells are more sensitive to ferroptosis. Ferroptosis has been observed in inflammation [13], ischemic reperfusion injury [14], hepatocellular carcinoma [15], and breast cancer [16]. However, there have been few studies focusing on the role of ferroptosis in GBM.

Heat shock protein 27 is a member of the small heat shock protein family and has been shown to be associated with ferroptosis [14]. Studies have shown that overexpression or phosphorylation of HSP27 can negatively regulate ferroptosis by inhibiting iron absorption and ROS production [14]. Acyl-CoA synthase long chain 4 (ACSL4) is one of the key enzymes in the lipid peroxidation pathway; it plays an important role in ferroptosis and its high expression promotes the occurrence of cellular ferroptosis [15].

Ubiquitinoids are members of the ubiquitin protein family and have similarities and differences resulting from ubiquitination modifications. Similar to ubiquitin, both are post-translational modifications, small ubiquitin-like modifier (SUMO) can be covalently attached to lysine side chains in multiple target proteins; the difference is that ubiquitination modification is a proteasome degradation process, while SUMO is not [16]. Targeting protein post-translational modifications (PTM) in the GBM environment may serve as a breakthrough point in the treatment of central nervous system malignancies.

In this study, we confirmed that decreased HSP27 expression promotes ferroptosis by promoting ferrous ion (Fe2+) absorption and reducing the SUMO-mediated modification of ACSL4, enhancing its stability. Therefore, targeting HSP27 may be an effective medical means to inhibit the progression of GBM.

## Materials and methods

### Brain tissue specimens and cell culture lines

Samples of human GBM and brain contusion tissues were obtained from the First Affiliated Hospital of Soochow University, Suzhou, China. This study was approved by the Ethics Committee of Soochow University. Human SHG-140 cell lines were obtained from the Department of Neurosurgery & Brain and Nerve Research Laboratory, The First Affiliated Hospital of Soochow University, Suzhou, China, after primary culture and identification by Short tandem repeat. U251 cell lines were obtained from the Cell Bank of the Chinese Academy of Sciences (Shanghai, China). Cells were cultured in DMEM (Gibco, Waltham, MA, USA) containing 10% fetal bovine serum (FBS).

### Bioinformatic analysis

We analyzed HSP27 expression and survival time in normal tissues and glioma tissues using the database of GBM and LGG (n = 701) in TCGA [17] and the database of mRNA-array (n = 301) in CGGA [18] were used as raw data for Bioinformatic information analysis.

### Antibodies

Anti-HSP27 (CST #50353) and anti-β-tubulin (CST #2146) were purchased from CST (Danvers, MA, USA); anti-ACSL4 (ab155282), anti-SUMO2/3 (ab81371), anti-FPN1 (ab239511), and anti-FTL (ab109373) were purchased from Abcam (Cambridge, UK);anti-FTH1 (A19544), anti-TF (A19130), and anti-TFRC (A5865) were purchased from ABclonal Technology (Woburn, MA, USA); horseradish peroxidase-labeled goat anti-mouse IgG and goat anti-rabbit IgG were purchased from ZSGB-Bio (Beijing, China); glutathione (S4606) and CHX (s7418) were purchased from Selleck Chemicals LLC (Houston, TX, USA); ferrostain-1 (M2698), erastin (M2679), and deferoxamine mesylate (M555) were purchased from Abmole Bioscience (Houston, TX, USA); and FAC (#SLCK8574) was purchased from Sigma-Aldrich (St Louis, MO, USA).

### Western blotting and immunofluorescence

Using a 10 mm cell culture dish, we added 200 μL lysate after the cells became full, which were then lysed on ice for 30 min. The cell lysate was collected and centrifuged at 4 °C 12,000 × g for 15 min. The supernatant was collected and the BCA (Beyotime, Shanghai, China) reagent was used to measure protein concentration. An equal amount of 8–12% SDS-PAGE was performed, the membrane was transferred to a polyvinylidene fluoride (PVDF) membrane, and it was blocked with 5% bovine serum albumin for 1 h at room temperature, and then left at 4 °C overnight. The primary antibody was recovered, TPST wash was used 3 times (5 min each time), and at room temperature secondary antibody incubation took place for 1 h after another 3 rounds of TBST wash, and then luminescent liquid was added dropwise for visualization. Cells were seeded in 12-well plates, cultured overnight, and fixed the next day with 4% paraformaldehyde and membrane-penetrating 0.5% Triton X-100. Then, the cells were incubated with 5% bovine serum albumin solution for 30 min at room temperature, incubated with primary antibody (1:100 dilution) overnight at 4 °C, and then incubated with a secondary antibody (1:100 dilution) for 1 h at room temperature. Staining with 4',6-diamidino-2-phenylindole was performed for the identification of nuclei. The slides were observed using a PerkinElmer UltraVIEW VOX (Waltham, MA, USA) fluorescence confocal microscope. We captured and analyzed images from 16 random fields of view at × 800 magnifications.

### Lentiviral transfection

Gene Chem (China) to produced two shRNA lentiviruses against HSP27, shHSP27#1: 9’-GATCACCATCCCAGTCACCTT-3’ and shHSP27#2: 9’-CCGATGAGACTGCCGCCAAGT-3’. The constructed lentiviral vector was transfected into cells followed by puromycin intervention. To identify stably transfected cells, western blotting analysis was used to determine transfection efficiency.

### Immunohistochemistry and hematoxylin and eosin staining

Paraffin-embedded tissues were sliced for staining. Paraffin wax sections were dewaxed and then incubated with 0.3% hydrogen peroxide at room temperature for 5–10 min to eliminate endogenous catalase activity. This was then rinsed with distilled water, nonspecific proteins were blocked with 5% goat serum (Solarbio Life Science, Beijing, China), the serum was discarded after 10 min, and not washed. An antibody was added and left at 4 °C overnight, with incubation of paraffin sections with ABC peroxidase and diaminobenzidine (ZSGB-Bio Ltd., Beijing, China) for nuclear staining using Mayer hematoxylin solution (Solarbio Life Science, Beijing, China) counterstainer. For hematoxylin and eosin (H&E) staining, the slides were counterstained using the H&E Kit (Solarbio Life Science, Beijing, China) after they were nuclear stained. Images were acquired using an inverted microscope (Olympus, Tokyo, Japan).

### Cell viability and proliferation assays

Cells were cultured at a density of 5000/well, 100 μL per well based on a 96-well plate, repeating three groups. After 24, 48, 72, and 96 h, 10% CCK8 reagent (Dojindo, Kumamoto, Japan) was added to each well, incubated in a 37 °C incubator for 2 h, and the absorbance value of 450 nm was measured to assess the proliferation capacity of cells. The control group and the experimental group each comprised 1,000 cells and seeded them in a 6-well plate, added 10% fetal bovine serum in 5 ml, and the solution was changed every 3 days. After two weeks, 4% of the cells were fixed, crystal violet stained, and Image J was used to count colonies with more than 100 cells. Proliferation was assessed using the EdU incorporation assay according to the manufacturer’s protocol (Beyotime, Shanghai, China). Briefly, EdU was incorporated into proliferating cells and detected through a catalyzed reaction with a fluorescently labeled azide. Labeled cells were examined under fluorescence microscopy, and the number of EdU-positive cells was counted in three independent experiments. According to the Calcein/PI Cell Viability/Cytotoxicity Assay Kit (Beyotime, Shanghai, China), cells were seeded in a 6-well plate, and after the cells had a suitable degree of fusion the supernatant was aspirated and the PBS was washed once. One ml of working solution was added to each well and then incubated at 37 °C for 30 min without light. Then, the staining results were observed under a fluorescence microscope (Calcein AM is green fluorescence, Ex/Em = 494/517 nm; PI is red fluorescence, Ex/Em = 535/617 nm).

### LDH release

Cell mortality was determined using a lactate dehydrogenase cytotoxicity assay kit (Beyotime, Shanghai, China). According to the instructions, the absorbance value of each well was determined at 490 nm, and the mortality rate was calculated according to the formula: cytotoxicity or mortality (%) = (treatment sample absorbance − sample control well absorbance) / (absorbance of the maximum enzyme activity of cells—sample control well absorbance) × 100%.

### Iron assay

An Iron Assay Kit (ab83366, Abcam, Cambridge, UK) was used for chromatic determination of cellular iron levels. Per 106 cells or 10 mg of tissue, 100 μL iron assay buffer was added to ice for sufficient homogenization, centrifuged at 4 °C 16,000 × g for 10 min, and the supernatant was left for later use. Fifty μL samples were added to 96-well plates, replenished to 100 μL with iron assay buffer, 5 μL of assay buffer was added for divalent iron assays, and 5 μL iron reducer was added to total iron assays and incubated at 37 °C for 30 min. Then, 100 μL of Iron Probe was added per well, incubated at 37 °C for 60 min, and determined the absorbance value of 593 nm with a microplate reader. The concentration of well iron in the sample was calculated according to the standard curve.

### Lipid peroxidation assessment

Lipid Oxidation (MDA) test kits were purchased from Beyotime (Shanghai, China). According to the experimental instructions, we added cells or tissues, lysate or PBS on ice to fully homogenize, centrifuged at 4℃ 12,000 × g for 10 min, took the supernatant and set it aside. After determining the protein concentration, we mixed the 100 μL sample with the 200 μL working solution, heated it at 100 °C or in a boiling water bath for 15 min, cooled the water bath to room temperature, centrifuged at 1000 × g for 10 min, took the 200 μL supernatant in a 96-well plate, determined the absorbance value at 532 nm with a microplate reader, and measured the MDA content according to the standard curve.

### ROS assay

A Reactive Oxygen Species Assay Kit was purchased from Beyotime (Shanghai, China). According to the experimental instructions, the appropriate cells were laid in a 96-well plate, and after the cell adherent reached the drug action time, the supernatant was aspirated, gently washed three times with serum-free high sugar medium, diluted DCFH-DA according to 1:1000 serum-free medium, so that the final concentration was 10 μmol/L. We then added 200 μL working solution per well, incubated this for 25 min with light avoidance, aspirated the supernatant, washed three times, added 200 μL complete medium, and used 488 nm excitation wavelength, 525 emission wavelength to measure fluorescence intensity.

### Mitochondrial membrane potential assay

The mitochondrial membrane potential in SHG140 and U251 cells was detected using the JC-1 Assay Kit (Beyotime, Shanghai, China) according to the manufacturer’s protocol. Cells were stained with JC-1 working solution for 20 min at 37 °C in an incubator and analyzed using inverted fluorescence microscope. The intensities of red (excitation: 530 nm; emission: 590 nm) and green fluorescence (excitation: 485 nm; emission: 528 nm) were measured. Assays were performed in triplicate, and fluorescence intensities were calculated using ImageJ software.

### Immunoprecipitation (IP)

Protein stock solution, with anti-HSP27 or ACSL4 antibody bound to magnetic beads, was incubated overnight at 4 °C. The pellet was washed 3 times with lysis buffer, boiled in 1 × sodium dodecyl sulphate (SDS) sample buffer for 5 min, and proteins were separated using SDS-PAGE on an 8–12% gel. Western blot detection was performed using appropriate antibodies.

### Transmission electron microscopy

Cells were cultured evenly in a 10 mm dish; after the cells became full, 1 ml of 0.25% trypsin solution was added for digestion in a 37 °C incubator for 5 min, an equal amount of medium was added to terminate digestion, followed by centrifuging to obtain the cell pellet. For material fixation, we discarded the culture medium and added the electron microscopic fixative solution to fix it for 2–4 h, the cells were then seen at low centrifugation in the bottom of the tube as mung bean-sized cell clumps, 1% agarose wrapped, rinsed with 0.1 M phosphoric acid buffer (pH 7.4) 3 times for 15 min each time. Regarding post-fixation, 1% osmium acid and 0.1 M phosphoric acid buffer (pH 7.4) were combined at room temperature (20 °C) and fixed for 2 h, and rinsed with 0.1 M of phosphoric acid buffer (pH 7.4) 3 times for 15 min each time. For dehydration, tissues were sequentially inserted into 50%-70%-80%-90%-95%-100%-100% alcohol -100% acetone-100% acetone ascend dehydration for 15 min each time. To infiltrate, acetone 812 embedding agent 1:1 was used for 2–4 h, acetone 812 embedding agent 1:2 was then used for penetration overnight, pure 812 embedding agent was then used for 5–8 h, pure 812 embedding agent was poured into the embedding plate, the sample was inserted into the embedding plate after use of a 37 °C oven overnight. For embedding, 60 °C oven polymerization was utilized for 48 h. Slices were obtained using an ultra-thin slicer to create 60–80 nm ultra-thin sections. Staining was performed with uranium-lead double staining (2% uranium acetate saturated alcohol solution, lead citrate, each stained for 15 min), and slices were dried overnight at room temperature. Transmission electron microscopy was used for observation and acquisition of image analysis.

### Nude mouse intracranial xenograft model

Thymic female BALB/c nude mice (4 weeks, 20–22 g) were purchased from the Institute of Oncology, Chinese Academy of Medical Sciences (Beijing, China). In the new pathogen-free environment, after three days of acclimatization with food and sterile water, 5 × 104 SHG-140 cells with luciferase-encoded lentivirus (Gene Chem, Shanghai, China) were stereotactically injected into the skulls of mice (6 in each group). Tumor size was recorded on days 7, 14, 21, and 28 using the IVIS Spectroscopic Live Imaging System (PerkinElmer, Branford, CT, USA). Mice were sacrificed after 28 days, the mouse brains were removed and fixed using 4% paraformaldehyde, paraffin-embedded, and HE as well as IHC staining were performed. The animal research was carried out in accordance with internationally recognized norms and national regulations.

### Statistical analysis

All experimental data were replicated at least three times, statistically analyzed using GraphPad Prism 9 software, and the differences between two or more groups were examined by *t*-test, one-way analysis of variance (ANOVA), and Tukey, and Kaplan–Meier survival analysis was used to assess mouse survival time. P-values less than 0.05 were considered statistically significant.

## Results

### High expression of HSP27 suggests a poor prognosis

**Fig. 1:**
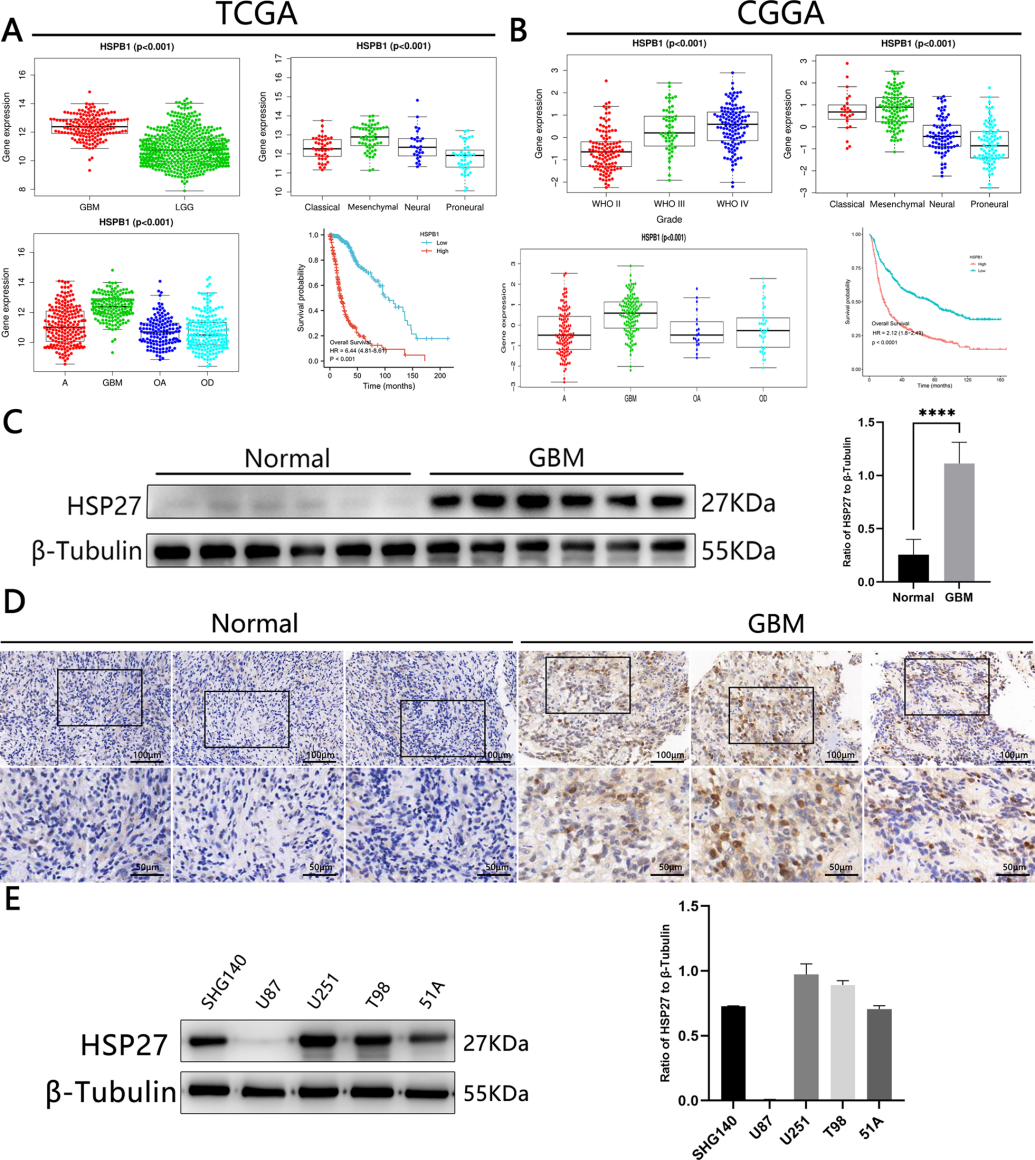
HSP27 is highly expressed in GBM and is associated with a poor prognosis.** A**, **B** The expression features in GBM subtypes were examined using the The Cancer Genoma Atlas and the Chinese Glioma Genome Atlas datasets. TCGA-seq and CGGA-seq data sets were used to estimate the correlation between HSP27 mRNA expression and tumor grade according to the World Health Organization. **C** Western blotting to detect the expression of HSP27 in normal tissues and tumor tissues. **D** Immunohistochemistry staining in normal and tumor tissues, the upper scale is 100 μm, and the lower scale is 50 μm. **E** Western blotting to detect the expression of HSP27 in different cell lines. Statistics are expressed as Mean ± S.E.M. ^NS^P > 0.05, ^****^P < 0.0001

First, we examined HSP27 mRNA levels of genes in human glioma samples for expression data from public datasets of the TCGA and CGGA databases (Fig. 1A, B). In contrast to brain contusion tissue, tumors express high levels of HSP27 mRNA, and this expression increases with the presence of gliomas. The expression of HSP27 in GBM is significantly higher than that in astrocytomas (A), oligodendrocyte astrocytomas (OA), and oligodendrocyte tumors (OD), and the expression of HSP27 mRNA in typical midline gliomas is higher than that of neurogliomas, classical gliomas, and gliomas. Kaplan–Meier analysis of TCGA and CGGA showed that the overall survival time of patients with a high expression of HSP27 in tumors was shorter than that of patients with low expression. At the same time, we obtained the same verification using IHC and western blotting of normal tissue and glioma tissue (Fig. 1C, D). We selected primary cells SHG140 [19] cultured from the Brain Nerve Research Laboratory of the First Affiliated Hospital of Suchow University and U251 purchased from the Shanghai Cell Bank for the following experiments (Fig. 1E). As shown above, HSP27 is highly expressed in GBM and has a poor prognosis.

### The silencing of HSP27 inhibits cell proliferation and reduces cell viability

**Fig. 2:**
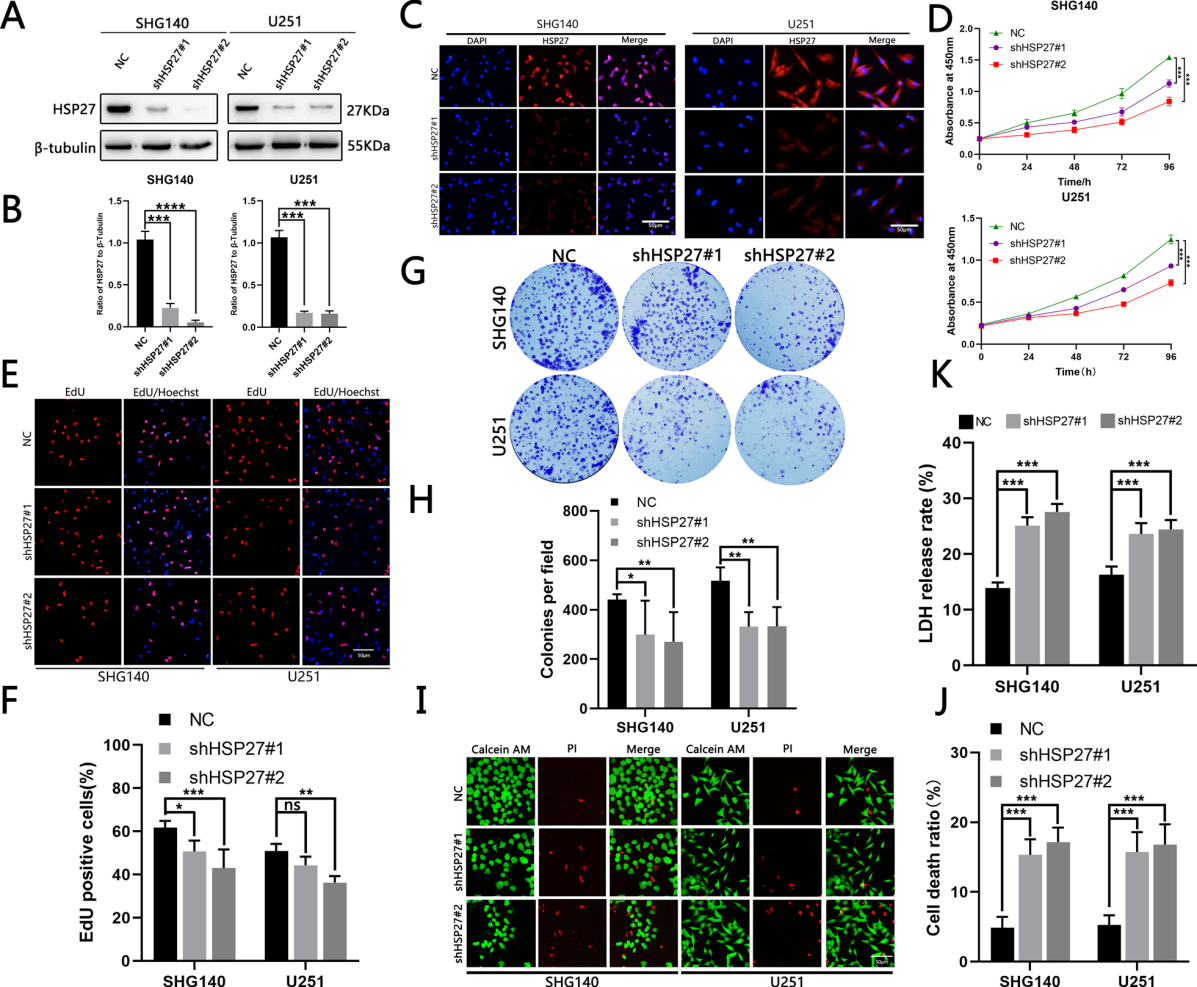
Silencing of HSP27 inhibits GBM cell viability and proliferation. **A**, **B** Western blotting verified shHSP27 transfection efficiency. **C** Immunofluorescence verified transfection efficiency, red fluorescence represents HSP27 expression, blue represents the nucleus, scale bar = 50 μm. **D** Growth curves for SHG140 and U251 cells before and after transfection, OD 450 nm at 24, 48, 72, and 96 h. **E**, **F** The EdU assay shows the fluorescence image and positive ratio before and after transfection of SHG140 and U251 cells. The red image represents EdU proliferation and the blue image represents EdU expression. **G**, **H** Colony formation experiments assessed the proliferation capacity of SHG140 and U251 cell lines before and after transfection of shHSP27, fixation of cells with 4% paraformaldehyde, and staining with crystal violet. **I**, **J** Fluorescence images of dead cells/live cells of the SHG140 and U251 cell lines before and after transfection of shHSP27, red represents dead cells, green represents living cells, scale bar = 50 μm. **K** LDH release in SHG140 and U251 cells before and after transfection with shHSP27. Student’s *t*-test for two-group comparison: ^NS^P > 0.05, ^*^P < 0.05, ^**^P < 0.01, ^***^P < 0.001,^****^P < 0.0001; one-way ANOVA for multi-group comparisons: ^NS^P > 0.05, ^*^P < 0.05, ^**^P < 0.01, ^***^P < 0.001, ^****^P < 0.0001

The TCGA and CGGA data suggested that high HSP27 expression is associated with a poor prognosis. We speculated that the knockdown of HSP27 would affect GBM cell viability. We designed and used two different shRNA lentiviruses to transfect SHG140 and U251 cell lines to reduce HSP27 expression. Knockdown effectiveness was confirmed by Western blotting and immunofluorescence staining (Fig. 2A–C). We then used CCK8 (Fig. 2D), EdU (Fig. 2E, F), and plate clones (Fig. 2G, H) to detect cell proliferation capacity, all suggesting that the proliferation capacity of cells transfected with shRNA is significantly lower than that of untransfected cells. Lactate dehydrogenase release experiments (Fig. 2K) and live/dead cell assays (Fig. 2I, J) suggested that the cell viability of transfected shRNA virus was decreased compared with that of the control group. As shown above, silencing HSP27 reduces GBM cell viability and inhibits their proliferation.

### *Inhibiting HSP27 results in an increase in ROS production mediated by Fe2* + *in cells*

**Fig. 3:**
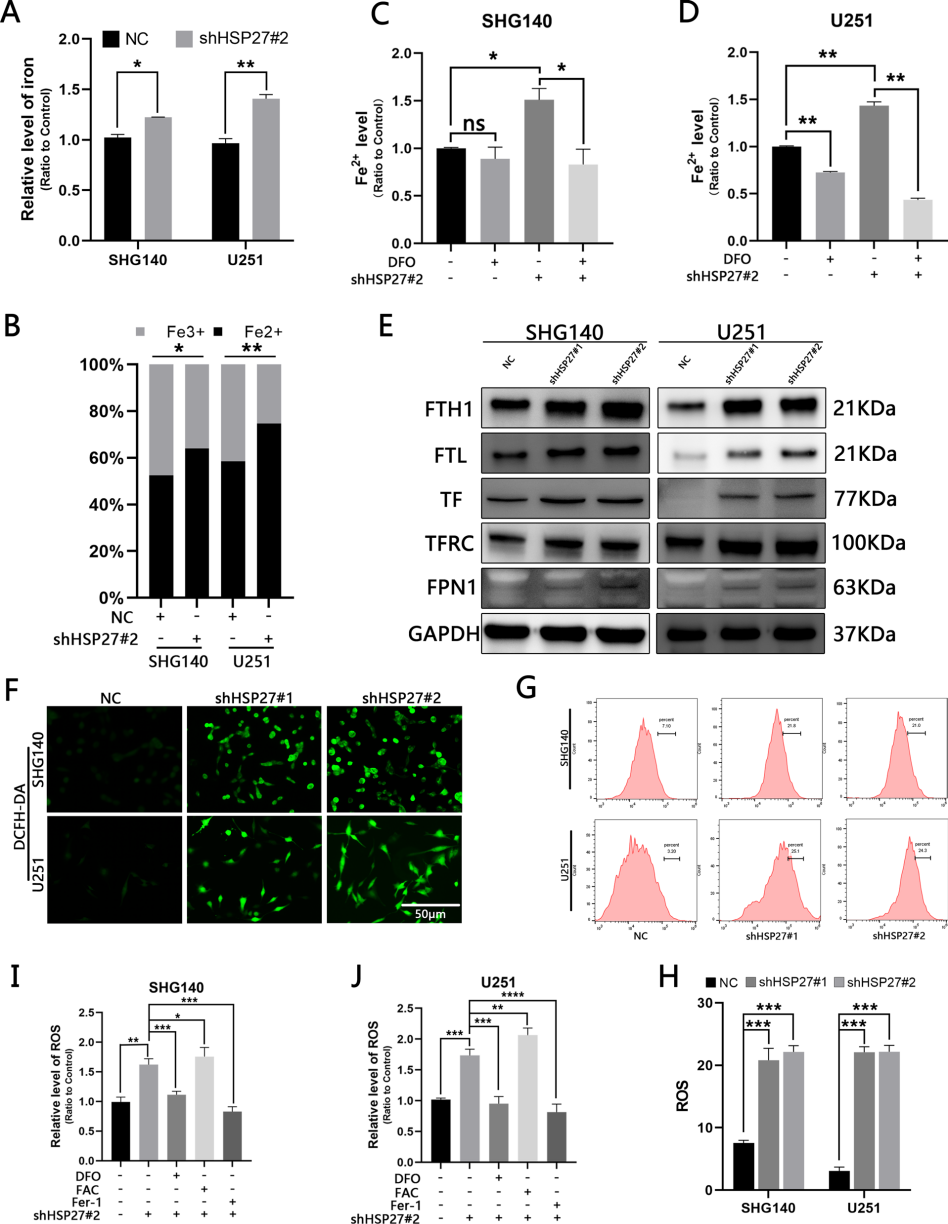
Silencing HSP27 increases cellular ferrous ion-mediated ROS production.** A**, **B** Total iron levels and ferrous ion ratios before and after shHSP27 transfection of SHG140 and U251 cell lines were determined by colorimetric methods. **C**, **D** Colorimetric determination of ferrous ion expression in different groups after DFO treatment. **E** Expression of iron-associated proteins in control groups and after lentiviral transfection. **F** Observation of ROS fluorescence intensity before and after transfection under fluorescence microscopy, DCFH-DA staining, scale = 50 μm. **G**, **H** Flow cytometry quantitatively detects ROS before and after transfection. **I**, **J** The microplate reader detects changes in ROS in different treatment and control groups. The statistical analysis showed ^*^P < 0.05, ^**^P < 0.01, ^***^P < 0.001, ^****^P < 0.0001, ^NS^P > 0.05

Studies have shown that HSP27 is associated with iron metabolism [13]. Therefore, we investigated whether the loss of HSP27 leads to changes in the iron content of cells. We found that the total iron content of the HSP27 silent group was significantly higher than that of the control group, and the divalent iron ions were the mainstay (Fig. 3A, B). Desferrioxamine (DFO) is an iron-chelating agent that can remove iron ions in cells and act on cells at a concentration of 500 μmol/L, which results in a significant decrease in intracellular ferrous ions (Fig. 3C, D). The process by which cells absorb iron is as follows: transferrin (TF) binds to extracellular Fe3 + to form a complex, which is then transported into the cell by a transferrin receptor (TFRC), which is mainly stored in the form of ferritin heavy chains and light chains (FTH1, FTL) [20]. Membrane iron transporter 1 (FPN1) is thought to be the only molecule that can transport iron within a cell to the outside of the cell [21]. We performed Western blotting analysis of the above iron-related molecules and found that silencing HSP27 leads to an increase in the expression of the above molecules (Fig. 3E). Therefore, we hypothesize that knockdown of HSP27 can lead to the accumulation of ferrous ions primarily by promoting iron absorption, rather than inhibiting iron output. Due to the excessive accumulation of ferrous ions within the cell, ROS is generated by the Fenton reaction. We measured the total ROS within the cells, measured the cellular ROS level with DCFH-DA, and found that the fluorescence intensity of the cells in the knockdown group was enhanced compared with the control group (Fig. 3F). The same changes (Fig. 3G, H) were observed by using flow cytometry quantification. To verify that HSP27 silencing correlates with ferroptosis, we applied Fer-1 (50umol/L, an inhibitor of ferrozomiosis), DFO, FAC (500umol/L, Ferric ammonia citrate, which increases intracellular iron content) to cells, and then quantitatively measured ROS changes, and the ROS level was significantly reduced under the action of DFO、Fer-1 and significantly increased during the action of FAC (Fig. 3I, J). Therefore, we conclude that silencing HSP27 leads to the accumulation of ferrous ions and induces ROS production through the Fenton reaction.

### Knockdown of HSP27 promotes ferroptosis in GBM cells

**Fig. 4:**
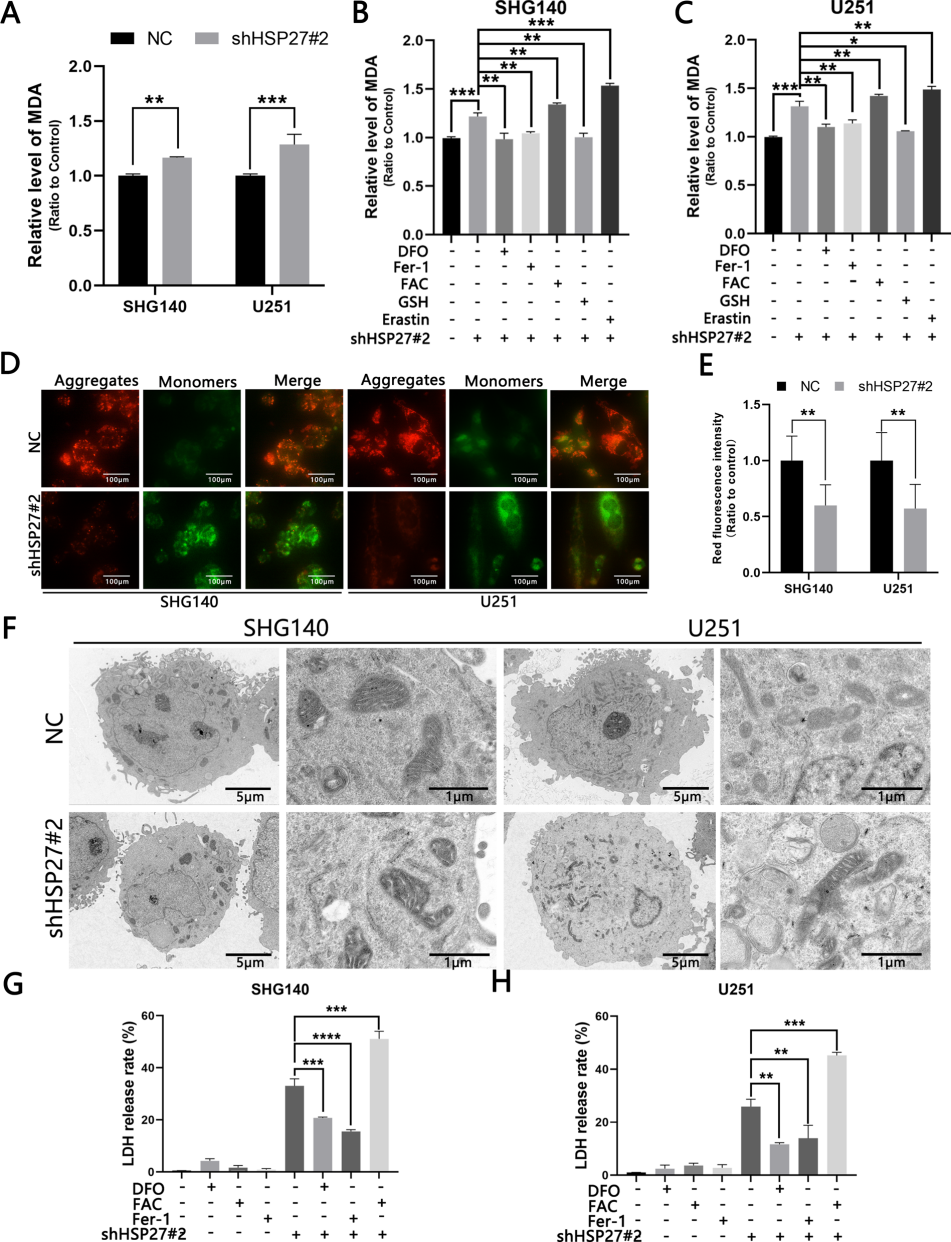
Knockdown of HSP27 promotes ferroptosis in GBM cells. **A**, **B** JC-1 detects mitochondrial potential changes at a scale of 100 μm. **C**–**E** Transfection with lentivirus and changes in MDA levels under different drug treatments. **F** Transmission electron microscopy (TEM) facilitates observation of changes in mitochondrial morphology before and after lentiviral transfection. **G**, **H** Detection of LDH release rates under different treatment methods. The statistical analysis showed the following: ^*^P < 0.05, ^**^P < 0.01, ^***^P < 0.001, ^****^P < 0.0001

ROS can lead to intracellular arachidonic acid (AA), adrenal acid (AdA) peroxide, and the peroxidation end products mainly include malondialdehyde (MDA) and 4-hydroxynenoal (4-HNE). We performed MDA detection on cells in different treatment groups and found that the knockdown group was elevated compared with the control group, and the DFO reversed the elevation of the knockdown group. The intracellular MDA content was significantly increased (Fig. 4A–C) under the action of ferric acid citrate (500 μmol/L), it can increase the content of ferrous ions in cells, which indicated that the increase in MDA was iron-dependent. To verify whether HSP27 knockdown caused ferroptosis we used ferrostatin-1 (Fer-1), a small molecule scavenger of free radical substances involved in lipid peroxidation. After we treated the cells with Fer-1 (50 μmol/L), we found a decrease in MDA, while the opposite changes occurred in the treatment group using the ferroptosis inducer Erastin (50 μmol/L). At the same time, we observed a decrease in MDA content under the action of GSH, which is also consistent with the characteristics of iron death oxidative stress (Fig. 4A–C). Another sign of ferroptosis is a change in the mitochondria. We used JC-1 to detect intracellular mitochondrial potential levels. Normal cells in the mitochondria exist mostly as aggregates, in JC-1 staining they appear as red fluorescence, and when the cells experience ferroptosis, the cellular mitochondria are subjected to oxidative stress of ROS resulting in mitochondrial concentration, membrane density increase, potential reduction, and other changes; the mitochondria are changed from aggregates to monomers, which mainly manifests as green fluorescence. shHSP27#2-transfected SHG140 and U251 cells showed a decrease in red fluorescence and an increase in green fluorescence, indicating that the knockdown group was lower than the control group (Fig. 4D, E). At the same time, we used transmission electron microscopy to observe the morphological changes of the mitochondria in the knockdown group and the control group, and found that the mitochondria of the knockdown group did appear to be concentrated and the membrane density increased (Fig. 4F). The ultimate goal of cellular ferroptosis is cell death and lactate dehydrogenase is released after cell death. We tested different treatment groups and control groups, and found that DFO can significantly reduce cell mortality, while the FAC treatment group showed the opposite change, and we also found that the knockdown of HSP27 increased the sensitivity of ferroptosis inducers (Fig. 4G, H). The above manifestations all indicate that the loss of HSP27 leads to iron-dependent ferroptosis in GBM cells.

### Silencing HSP27 promotes ferroptosis by reducing its SUMOylation of ACSL4 to enhance its stability

**Fig. 5:**
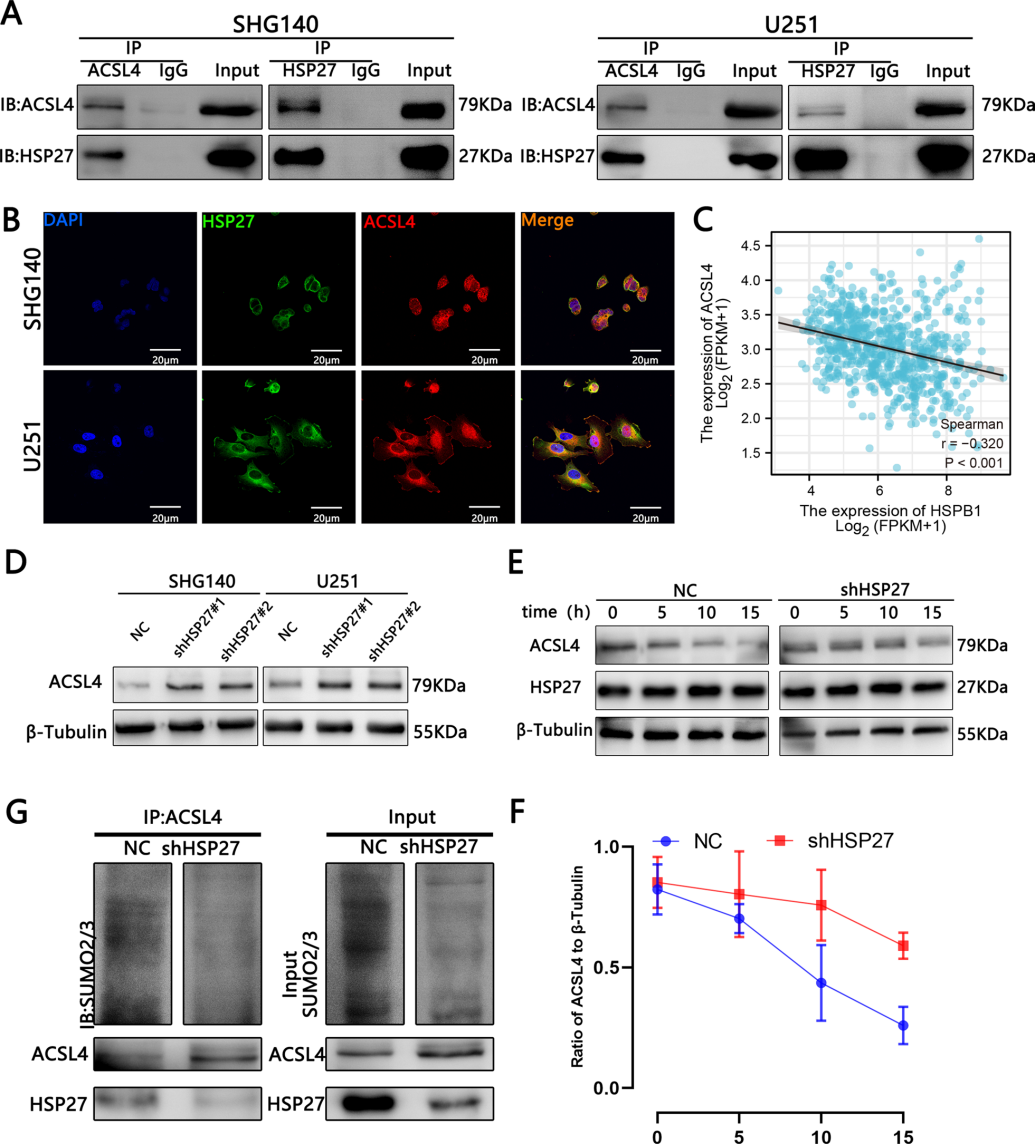
The interaction between HSP27 and ACSL4 in glioma cells. **A** Immunoprecipitation followed by western blot analysis revealed the interaction between HSP27 and ACSL4 in glioma cells. **B** Immunofluorescent images of HSP27 and ACSL4 expression in glioma cells. The nuclei were stained with DAPI. Images were captured by a laser confocal microscope. **C** Correlation analysis of TCGA database HSP27 and ACSL4. **D** Western blot detects changes in ACSL4 after HSP27 silencing. **E**, **F** After cycloheximide (20 μmol/L) treatment, the ACSL4 stability test was performed. **G** SUMOylation analysis of ACSL4 in the control group and HSP27 in the silence group

ACSL4 is one of the key enzymes in the lipid peroxidation process of ferroptosis, and its high expression promotes the occurrence of ferroptosis [15]. HSP27 and ACSL4 are ferroptosis-related molecules. Their respective effects on ferroptosis have also been studied. However, the associated ferroptosis link between HSP27 and ACSL4 has not been elaborated. Studies have shown that SENP1 can modulate the inflammatory signal A20, which interacts with ACSL4 and SLC7A11 to regulate ferroptosis in lung cancer cells [22]. Therefore, we suspected that glioma cells transfected with HSP27 lentivirus are associated with a SUMO-related association with ACSL4. We first performed COIP and fluorescence confocal experiments on HSP27 and ACSL4 in SHG140 and U251 cell. The results showed that there was a clear direct correlation (Fig. 5A, B). Furthermore, TCGA and western blot analyses suggest a negative correlation between HSP27 and ACSL4 (Fig. 5C, D). This also lays the foundation for the following experiment. It fits the study that high expression of ACSL4 promotes ferroptosis. Next, we treated the NC and shHSP27 groups with actinomycone (CHX, 20 μmol/L) for 0, 5, 10, and 15 h, and then performed western blot analysis for ACSL4 stability. The results showed that with the increase of CHX treatment time, the expression of ACSL4 in the control group was significantly reduced, especially at 10 and 15 h, and was statistically significant. A decreased in ACSL4 expression was also observed in the HSP27 knockdown group, but not as pronounced as in the control group (Fig. 5E, F). To further validate the hypothesis, we performed IP experiments on SUMO2/3 of ACSL4 in the control group and the knockdown group. The results showed that the SUMO2/3 level of ACSL4 in the knockdown group was reduced (Fig. 5G). In summary, there is a direct correlation between HSP27 and ACSL4 and knockdown of HSP27 may inhibit the SUMOization of ACSL4 and promote ACSL4 stability to promote the occurrence of ferroptosis.

**Fig. 6:**
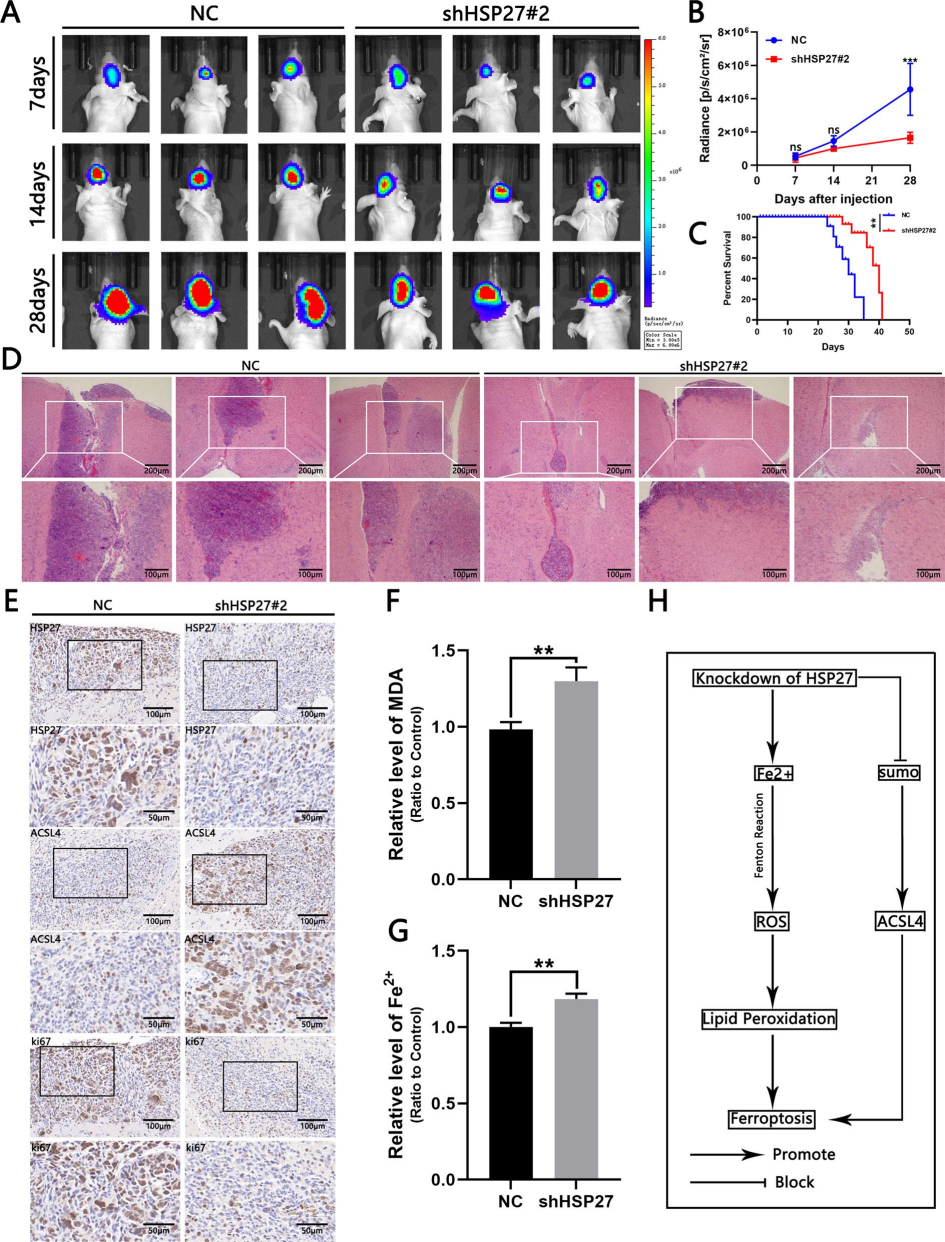
Knockdown of HSP27 inhibits tumor growth in vivo. **A** Fluorescent images of intracranial tumor size in mice on days 7, 14, and 28 in the control and experimental groups. **B** Quantitative analysis of fluorescence images. **C** Mouse survival curves of control and experimental groups, n = 6. **D** H&E slice of mouse intracranial tumor, scale bar = 100 μm. **E** IHC image of anti-HSP27, Ki67 and ACSL4 tumor slices, scale = 100 μm. **F, G** Comparison of Fe2 + and MDA levels of xenograft samples in the control and experimental groups. **H** Schematic of HSP27 inducing ferroptosis in GBM. Student’s *t*-test for two-group comparisons: *p < 0.05, **p < 0.01, ***p < 0.001; log-rank test: *p* < 0.05

### Silencing HSP27 reduces intracranial tumor growth

To study the effect of HSP27 on GBM, we transplanted SHG140 cells transfected with negative control virus and shHSP27#2 lentivirus into small female nude mice brains to establish an intracranial in situ xenograft model. Tumor growth was observed by bioluminescence imaging on days 7, 14, and 28 after the transplantation of cells. The results showed that the growth rate of intracranial tumors in the experimental group was significantly lower than that in the control group (Fig. 6A, B). The survival time of mice in the experimental group was also significantly higher than that in the control group (Fig. 6C). In addition, H&E staining of nude mouse brain slices showed differences between the two groups (Fig. 6D). Brain slice IHC showed that the intensity of immunostaining of ki67, a nuclear proliferation marker, was lower than that of the control group, and ACSL4 were increased (Fig. 6E). In addition, the tissue MDA and Fe2 + expression in the experimental group were higher than those in the control group (Fig. 6F, G). In summary, the knockdown of HSP27 may inhibit the growth of GBM cells through ferroptosis.

## Discussion

Cell death is a complex and diverse process, and the forms of death include apoptosis, autophagy, necrosis, and pyroptosis [23]. Ferroptosis is a newly discovered form of programmed cell death, mainly based on iron-dependent lipid peroxidation [10]. Iron metabolism is now considered a key metabolic marker of cancer [24]. It has been confirmed that iron metabolism in tumor cells is more active than normal cells [24]. In this study, we analyzed the TCGA and CGGA databases and found that high expression of HSP27 was associated with poor prognosis and malignant glioma. In vitro experiments have shown that the knockdown of HSP27 can affect its viability and inhibit cell proliferation, promote Fe^2+^ absorption, inhibit ACSL4 SUMOization, and enhance ACSL4 stability leading to the accumulation of cellular lipid peroxidation, which ultimately leads to cellular ferroptosis. In vivo experiments have shown that the knockdown of HSP27 can significantly slow down the growth rate of intracranial tumors in mice and significantly prolong the survival time. Therefore, studying this gene-related targeted drug may treat GBM by inducing ferroptosis.

HSP27 has long been thought of as a chaperone involved in the regulation of the cytoskeleton of tissues [25] or stabilization of abnormally folded proteins to prevent aggregation [26]. HSP27 regulates a variety of biological processes in tumor cells and studies have shown that glioma cells express high levels of HSP27 compared to normal cells, which mediates secreted protein acidic and rich in cysteine (SPARC)-induced changes in glioma migration and invasion [27]. HSP27 promotes glioma cell proliferation through SIRT2-mediated G6PD activation [28]. Schultz found that inhibition of HSP27 alone, or in combination with pAKT inhibitors, may be an effective treatment to inhibit SPARC-induced glioma cell invasion and survival [29]. Recent studies have shown that HSP27 is a novel modulator of ferroptosis in cancer cells [14]. The relationship between HSP27 and ferroptosis has been reported, and overexpression of HSP27 inhibits the ferroptosis of erastin-induced HeLa, U2OS, and LNCaP cells, and inhibits HSP27 expression to promote ferroptosis [10, 30, 31]. HSP27 overexpression has been shown to protect glioblastoma cells from erastin-induced ferroptosis, while HSP27 depletion promotes erastin-induced glioblastoma ferroptosis [14].

Iron is a trace element that is essential for almost all cell survival processes, but excessive accumulation can lead to cell death [32, 33]. For example, ferroptosis is a type of cell death caused by the accumulation of iron-dependent lipid ROS, either by reducing substances or iron-chelating agents [10] and other inhibition of ferroptosis occurs. The specific mechanisms underlying ferroptosis remain unclear and there may be a role of alteration in the activity of iron-dependent enzymes [5]. Previously, HSP27 was thought to be a negative regulator of iron accumulation and uptake within fibroblasts and heart cells [34–36]. It remains difficult to inhibit GBM cells with chemical drugs alone [37]. There is an urgent need to explore new therapeutic targets to improve the effectiveness of GBM treatments. Inducing ferroptosis in tumor cells by increasing iron levels in GBM cells in various ways could be a new breakthrough point [38]. Ubiquitination and SUMOization are post-translational modification. Ubiquitination modification involves proteasome degradation, mainly by degrading one or more proteins to achieve the purpose of action. SUMOization does not involve the process of protein degradation. SUMOization levels are significantly upregulated in GBM, and SUMOization gene silencing inhibits DNA synthesis and cell growth, reducing cell survival [39]. Studies have shown that SUMO is involved in DNA damage [40], the cell cycle [41], and other related protein modifications. Assessment of SUMOization status may ultimately help predict and personalize GBM treatment in the future [42]. Here, we reaffirm previous research that silencing HSP27 promotes ferroptosis in GBM cells; at the same time, a new ferroptosis signaling pathway has been discovered, and silent HSP27 may inhibit the SUMOization of ACSL4 and promote ACSL4 stability to promote the occurrence of ferroptosis.

In this experiment, although the stability of ACSL4 was detected by CHX treatment and the change of sumo2/3 after HSP27 knockdown was detected by IP. It is proved that the SUMOization of ACSL4 is affected by its interaction with HSP27. But the experimental process seems to be inadequate. To further verify how HSP27 regulates SUMOization of ACSL4. The establishment of mutant colonies can be detected by predicting the sumo site of ACSL4 and by mutating, sequencing, and performing western blot analysis on this site. Effects of HSP27 on SUMO after ACSL4 locus mutation detected by using SUMO assay [43]. Finally, it can be determined that HSP27 regulates the position of ACSL4 at a certain site, enhancing the stability of ACSL4 protein. Interestingly, we found that HSP27 expression was very low to even not expressed in U87 cells. This is contrary to the previous results. We reviewed the CCLE database and showed that the expression of HSP27 in the same cell line was also different. Moreover, cells may have genetic mutations in an infinite number of passages. It also suggests that malignant tumor cells are extremely unstable. It may be difficult to eliminate tumors with a single treatment approach, and finding new treatments is currently needed. The new discovery suggests that the HSP27/SUMO/ACSL4 axis may inhibit glioma by ferroptosis.

## Conclusion

Overall, we show that the knockdown of HSP27 promotes Fe2 + absorption and promotes ROS production by inducing the Fenton reaction, while excessive ROS accumulation can trigger lipid peroxidation and a decrease in mitochondrial membrane potential, leading to ferroptosis. Moreover, we found an inverse correlation between HSP27 and the lipid peroxidase ACSL4, a key enzyme, and knockdown of HSP27 that inhibits the SUMOization of ACSL4 and enhances its stability. ACSL4 is known to cause ferroptosis by shaping lipid composition [15]. Its high expression promotes ferroptosis in GBM cells [44]. Therefore, HSP27/SUMO/ACSL4 shaft and iron upregulation may be a new direction for the treatment of GBM.

## Data Availability

The datasets generated and/or analyzed during the current study are available in The Cancer Genome Atlas (TCGA) data portal (https://tcga-data.nci.nih.gov/tcga/) and Chinese Glioma Genome Atlas(CGGA) database (http://www.cgga.org.cn/). We hereby undertake that all data and materials are available.
